# Haplotype Heritability Mapping Method Uncovers Missing Heritability of Complex Traits

**DOI:** 10.1038/s41598-018-23307-4

**Published:** 2018-03-21

**Authors:** Masoud Shirali, Sara A. Knott, Ricardo Pong-Wong, Pau Navarro, Chris S. Haley

**Affiliations:** 1 MRC Human Genetics Unit, MRC Institute of Genetics and Molecular Medicine, University of Edinburgh, Edinburgh, EH4 2XU UK; 2 Institute of Evolutionary Biology, University of Edinburgh, Edinburgh, EH9 3FL UK; 3 The Roslin Institute and R (D) SVS, University of Edinburgh, Easter Bush, Midlothian, EH25 9RG UK

## Abstract

We propose a novel approach to analyze genomic data that incorporates haplotype information for detecting rare variants within a regional heritability mapping framework. The performance of our approach was tested in a simulation study based on human genotypes. The phenotypes were simulated by generating regional variance using either SNP(s) or haplotype(s). Regional genomic relationship matrices, constructed with either a SNP-based or a haplotype-based estimator, were employed to estimate the regional variance. The results from the study show that haplotype heritability mapping captures the regional effect, with its relative performance decreasing with increasing analysis window size. The SNP-based regional mapping approach often misses the effect of causal haplotype(s); however, it has a greater power to detect simulated SNP-based-variants. Heritability estimates suggest that the haplotype heritability mapping estimates the simulated regional heritability accurately for all phenotypes and analysis windows. However, the SNP-based analysis overestimates the regional heritability and performs less well than our haplotype-based approach for the simulated rare haplotype-based-variant. We conclude that haplotype heritability mapping is a useful tool to capture the effect of rare variants, and explain a proportion of the missing heritability.

## Introduction

Genome-wide association studies have detected numerous common variants that affect complex quantitative traits. There is, however, a large proportion of the genetic variance that remains to be explained. For human adult height, for example, the heritability is around 0.80^1^ but single SNP analysis of 183,727 individuals explained only 12% of the trait heritability^2^ and joint multiple-SNP analysis of 253,288 individuals explained only one-fifth of human adult height heritability^3^. Some possible explanations for this missing heritability include common variants with small effect on the trait^4^, rare variants with large effects^5^ or imperfect linkage disequilibrium between the genotyped SNPs and the causal variants^6^. Low linkage disequilibrium is likely to be largely due to causal variants having lower minor allele frequencies than genotyped SNPs if they are subject to purifying natural selection^7,8^. This will result in the variance explained by the genotyped SNPs being lower than the variance explained by the causal variants.

Analytical approaches using multiple SNPs jointly (e.g. SKAT^9^ or regional heritability mapping^10^) have been shown to have more power in detecting both common and rare variants than single SNP mapping methods^11^. Multi-allelic markers such as haplotypes inferred from genotyped SNPs^12,13^, have been used to study the genetic structure of populations^14–16^ and also to detect un-genotyped causal variants^17–19^. Although, genotype imputation techniques have been presented to predict genotypes at variants that are not directly measured^20^, they are dependent on a reference panel that has been densely genotyped to identify shared haplotypes between the reference and the target populations^21,22^.

Nagamine *et al*.^10^ presented a regional heritability mapping method (RHM) that scans the genome using overlapping sliding windows of a given number of SNPs and includes in the analytical model both a whole genome relationship matrix and a regional genomic relationship matrix obtained from the SNPs from each region. In the present study we extend this idea, but use haplotype rather than SNP information to infer regional relationships amongst individuals and call this novel approach Haplotype Heritability Mapping (HHM). Additionally, we use haplotype blocks identified by recombination hotspots instead of windows determined by a fixed number of SNPs to facilitate meta-analysis of cohorts with different genotypic information.

The use of haplotype rather than SNP genotype information could potentially enhance the detection of rare variants that have effects on phenotypes, which are usually missed by standard mapping methods. Simulated data are used to develop and investigate the performance of the HHM approach and compare it to that of the RHM.

## Results

Results were obtained from populations simulated based on the haplotype frequencies observed in three Southern European cohorts: from the city of Split and islands of Vis and Korcula on the Dalmatian coast of Croatia^23^. Simulated phenotypes were generated as a combination of a regional genetic effect, a genome wide polygenic component, and a residual component. For the regional effect, a total of 20 distinct genomic regions were selected, and one of them in turn was used as causative region. Four different regional genetic architectures were simulated for each replicate in each region. Two of the simulated architectures were “SNP-based” with either one SNP (1SNP) or all SNPs (AllSNP) in the region being the causative variants; whereas, the other two architectures were “haplotype-based” with either one haplotype (1Hap) or all haplotypes (AllHap) in the region being causative. Whichever architecture was simulated, the total regional heritability contributed was the same and was set at 5% of the total variance. A total of ten population replicates were produced for each causative region and genetic architecture (hence 200 simulations for each genetic architecture). The genome wide polygenic component was simulated with each SNP in the genome outside the region of interest contributing to a genetic heritability of 25%. All simulated architectures were analysed using either RHM (where we used genotype information to construct the regional genomic relationship matrix (RGRM)) or HHM (where the RGRM was constructed from haplotype information). Analysis windows bounded by recombination hotspots were used that corresponded to the simulated ones or we used larger analysis windows containing the simulated ones. The results are presented for each genetic architecture, separately; and the mapping performance of the RHM (SNP-based analysis) and the HHM (haplotype-based analysis) were compared within architectures, in terms of power (by looking at likelihood ratio test (LRT)), and accuracy of estimation by looking at regional heritability (RH) estimates.

### SNP-based architectures

**Figure 1:**
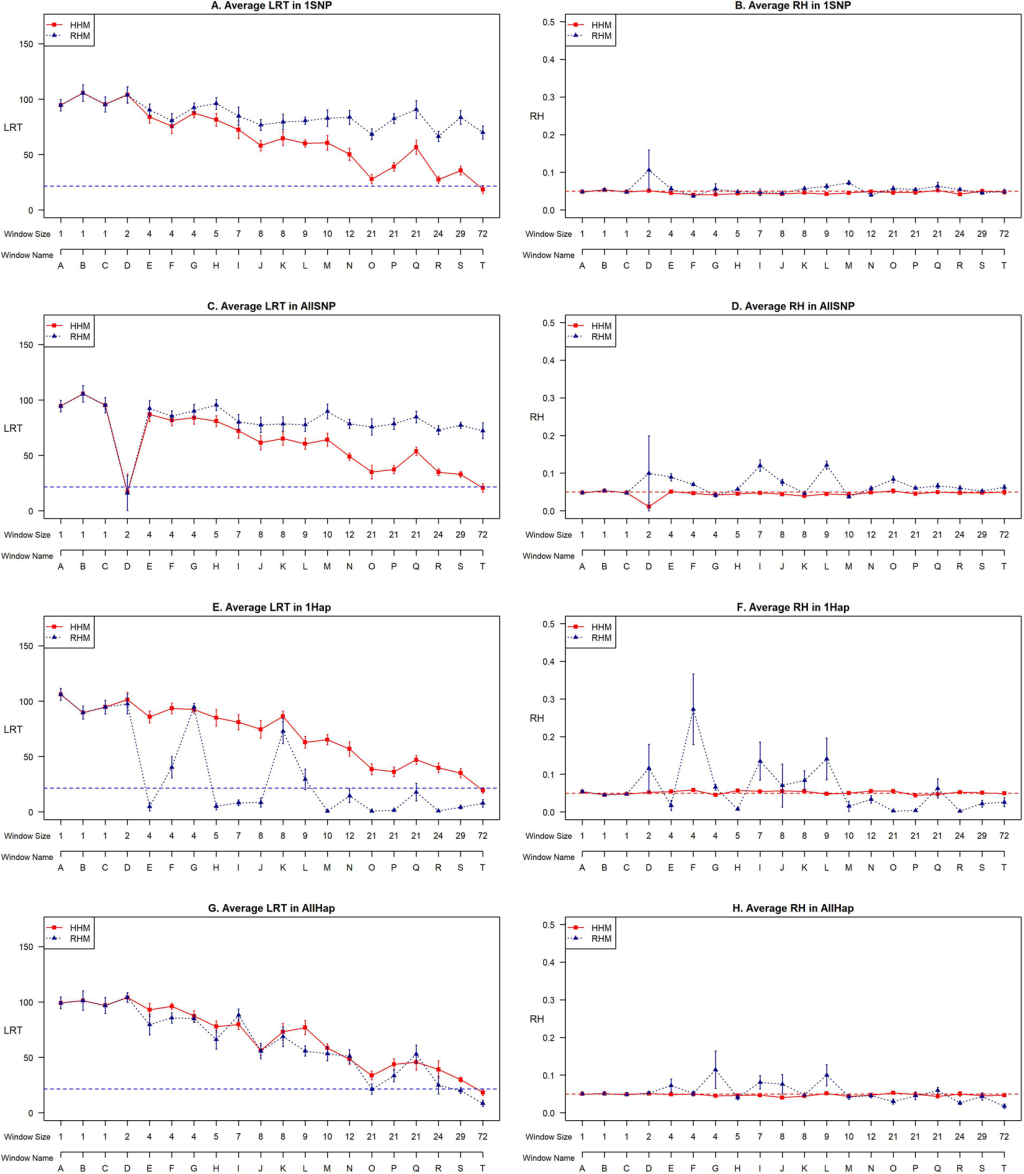
Average LRT (**A**,**C**,**E**,**G**) and RH (**B**,**D**,**F**,**H**) for the 20 regions analyzed ordered by window size measured as number of SNPs for the 1SNP, AllSNP, 1Hap and AllHap simulations using a threshold of 5 cM/Mb to define block boundaries for both the HHM and the RHM methods.

The average LRT and RH (over the 10 replicates) for the 1SNP and the AllSNP simulation scenarios are presented in Fig. 1A–D for both the RHM and the HHM analysis approaches, when the analysis windows correspond to the simulation regions (or ‘blocks’, with block boundaries being hotspots with at least 5 cM/Mb). For both the 1SNP and the AllSNP scenarios, the RHM detected all the regional effects and the HHM detected the effects in more than 94% of the individual replicates. An increase in number of SNPs in the window resulted in a decrease in the LRT especially for the HHM approach, leading to the mean LRT being borderline significant at the genome wide 5% level for the largest window (with 72 SNPs)(see Supplementary Tables S3 and S5). When re-analysing the data using analysis windows based on boundaries of 10 cM/Mb recombination, i.e. larger window boundaries than those used in the simulation, similar results to those obtained when using 5 cM/Mb analysis windows were observed (see Supplementary Figures S1A–D). To understand the effect of increasing the analysis window size from using a 5 cM/Mb (T5) to a 10 cM/Mb (T10) based window, we calculate the proportion of average LRT and RH estimated by T10 to the average estimated by T5 in both the HHM and RHM methods and plotted them against the ratio of the number of SNPs in the T10 window to those in the T5 window. The results demonstrate that the averaged LRT estimated by both methods are lower with the increased window size in both 1SNP and AllSNP scenarios (see Supplementary Figure S2A and C). Also, the averaged RH estimated by RHM was lower with the increased window size but in HMM the averaged RH remained constant (see Supplementary Figure S2B and D).

### Haplotype-based architectures

The 1Hap and the AllHap scenarios represent situations where the causal variants were not in strong LD with any single SNP. In the 1Hap scenario, a single causal variant was associated with one haplotype within the region, so that one ‘haplotype allele’ had an effect whereas the others did not. In the AllHap scenario there were multiple causal variants associated with all haplotype alleles such that each haplotype allele in the region had a randomly selected effect on the trait, drawn from a normal distribution. The average LRT and RH over the ten replicates in the casual region for the HHM and the RHM are presented in Fig. 1E–H for the 1Hap and the AllHap scenarios when using 5 cM/Mb analysis windows. The HHM method yielded similar average LRTs to those observed for the HHM method in the SNP-based architectures, and HHM performance declined with increasing analysis window size (see Supplementary Tables S7 and S9). In the 1Hap scenario, RHM yielded very inconsistent results with some of the smaller windows giving an average LRT similar to the HHM approach, for example in region G, whilst RHM did not detect an effect in other windows of the same size, such as the windows in regions E and F. In the AllHap scenario, HHM and RHM performed similarly, with a much faster drop in average LRT for the RHM than its performance seen in the SNP-based architectures as the window size (number of SNPs) increased. Similar results were obtained for the analysis based on 10 cM/Mb boundary (which is a larger window boundary than that used in the simulation) and the results are presented in Supplementary Figure S1E–H. The results comparing averaged LRT and RH obtained with the T10 and T5 windows, demonstrate that the averaged LRT estimated by both methods decreased with the increased the window size in both 1Hap and AllHap scenarios (see Supplementary Figure S2E and G). In both 1Hap and AllHap, the averaged RH estimated by RHM also declined with the increased window size but in HMM the averaged RH remained constant (see Supplementary Figure S2F and H).

In all SNP-based and haplotype-based architectures, comparing the estimated RH from the RHM and the HHM illustrated that the HHM gave results consistent with the simulated value of 0.05 across all simulated regions and scenarios (Figures 1B, D, F and H, and S1B,D,F and H). The RH estimates from the RHM were less accurate than those from the HHM, frequently overestimating the regional variance. In RHM, the mean of estimated RH over all scenarios, was slightly inflated (average RH = 0.060) and the mean squared error of RH (variance of estimated RH from 0.05 = 0.0063) was higher than the HHM (0.0002) (Supplementary Tables S4, S6, S8, and S10). The standard error of estimated RH over all scenarios was lower for the HHM (0.024) compared to the RHM (0.035) (Supplementary Figure S3).

**Figure 2:**
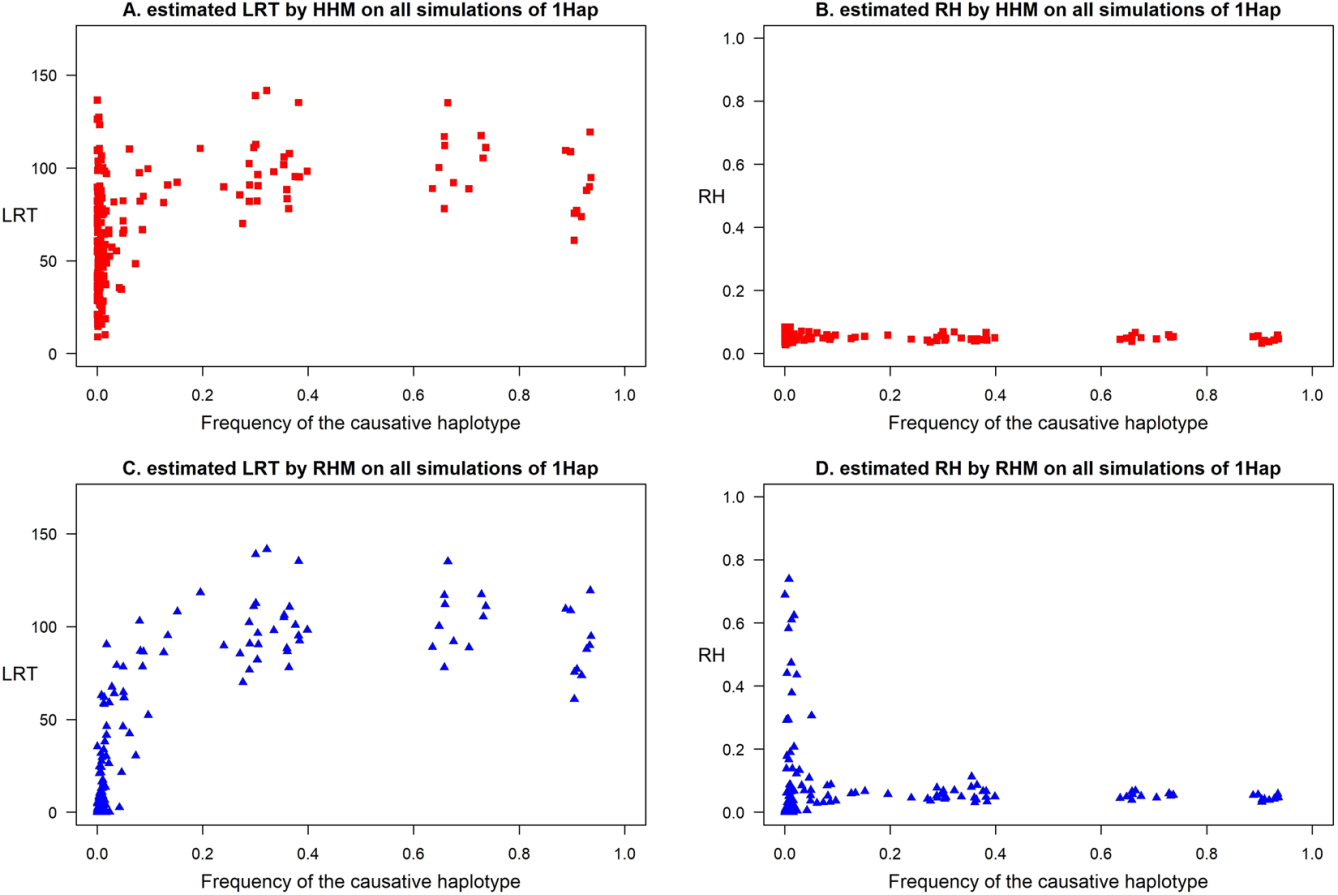
The relation between the frequency of the causal haplotype for the 1Hap simulation and estimated LRT (**A** and **C**) and RH (**B** and **D**) over all the regions analyzed using a threshold of 5 cM/Mb to define block boundaries.

**Figure 3:**
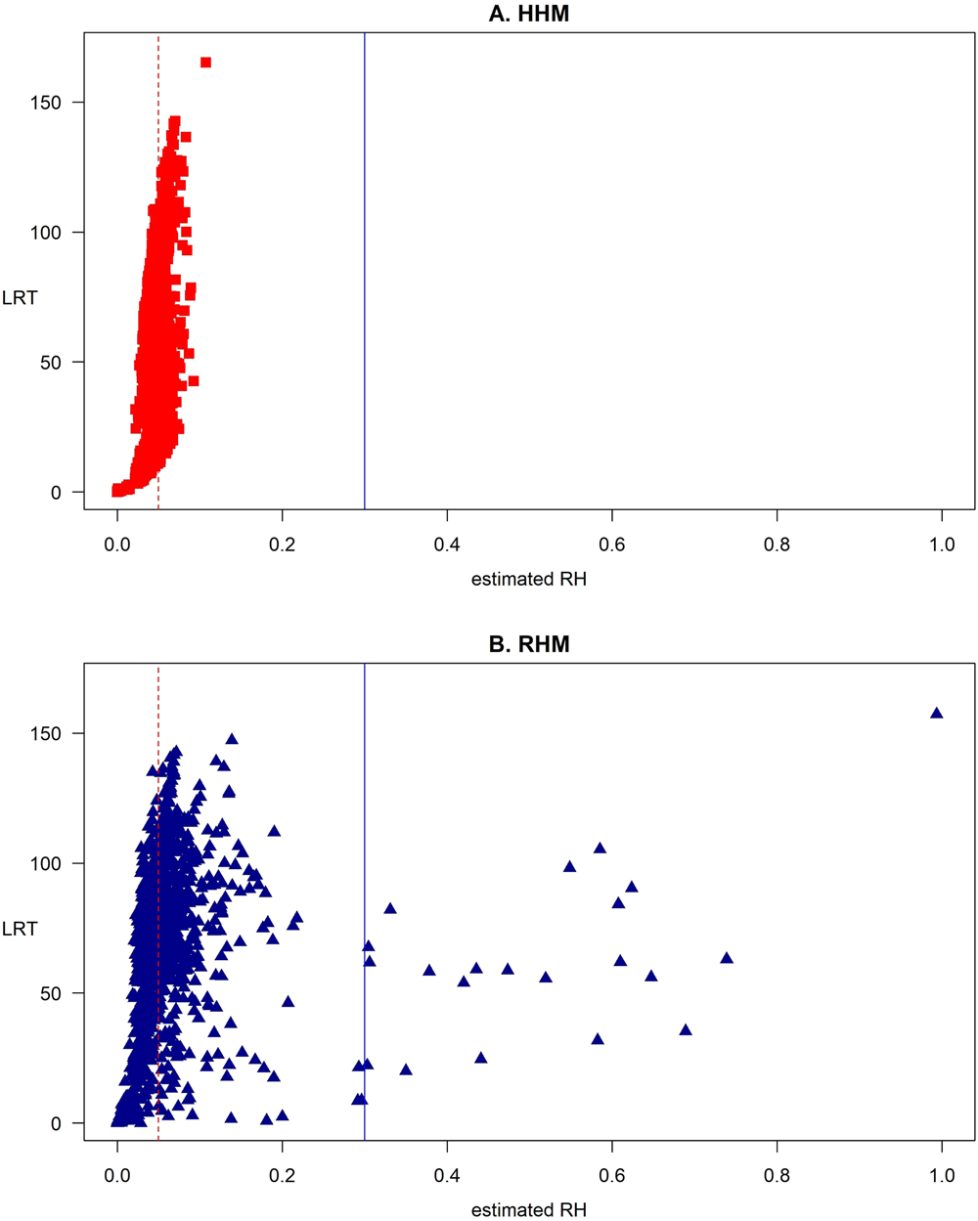
Plot of estimated RH against LRT for the four simulated scenarios (1SNP, AllSNP, 1Hap and AllHap) by using the HHM (**A**) and the RHM (**B**) for all replicates of the simulated regions.

Based on the obtained LRT and RH estimations in all scenarios, the most dramatic differences between HHM and RHM were for the 1Hap scenario (Fig. 1). To explain further the differences in performance between the HHM and the RHM for the 1Hap scenario, we studied the estimated LRT and RH in each region and replication of the 1Hap scenario. The estimated LRT and RH by each method were plotted against the frequency of the causal haplotype for each individual replicate (Fig. 2). Comparing the estimated LRT from the two methods showed that when the causative variant was a low frequency haplotype, HHM detected the effect of regions with higher test statistics than RHM (Fig. 2A and C). The estimated RH by RHM was strongly influenced by the frequency of causal haplotype while the estimates by HHM seemed to be independent of the frequency (Fig. 2B and D). In all the simulation scenarios, the HHM approach provides more accurate estimation of regional heritability for all the genetic architectures simulated than the RHM approach (Fig. 3). Comparisons between the HHM and RHM approach in estimated RH against LRT for the simulated scenarios (1SNP, AllSNP, 1Hap and AllHap) for all replicates of the simulated regions are presented in Supplementary Figure S4.

## Discussion

We proposed the novel method of Haplotype Heritability Mapping (HHM) to detect rare variants by estimating the haplotype block regional heritability through fitting a haplotype-based regional genomic relationship matrix in the model. In addition, we proposed to use haplotype blocks as units of analysis in both Haplotype and Regional (SNP-based) heritability mapping approaches, which should facilitate meta-analysis across different cohorts or populations. Our simulation study demonstrated the advantages of HHM in detecting causal regions when causal variants were based on haplotypes rather than SNP genotypes as would be the case when novel trait-associated variants have arisen in that haplotype and are not in strong LD with individual variants. The HHM approach also provides more accurate estimation of regional heritability for all the genetic architectures simulated than the RHM approach. The SNP based RHM approach performed best when analysing phenotypes simulated based on one SNP or the combined effects of single SNPs. Analysing the individual haplotype blocks used for the simulation gave higher average LRT than analysing larger windows comprising two or more SNPs in the blocks. Thus, in general the power to detect an effect was maximal when the analysis approach matched the model used to simulate the data both in terms of how the phenotypic effect was generated and the size of the haplotype with which it was associated. Irrespective of this, using either block definition, longer block sizes (in terms of the number of SNPs) gave lower mean LRT for both analyses but especially for HHM. The RH estimates were not affected by window size.

The RHM and HHM methods differ in their estimation of the regional relationship matrix. Both approaches weight the contribution from each allele (SNP or haplotype) to the regional relationship matrix, such that individuals sharing rare alleles are estimated to be more closely related than those sharing common alleles. This effect will be more extreme considering haplotypes, where very rare alleles can occur, rather than the combined effects of many SNPs. Additionally, individuals sharing many SNP genotypes in a haplotype block, and therefore relatively highly related under RHM, may differ in their haplotypes and be effectively unrelated using the HHM approach. These two effects mean that our haplotype-based regional genomic relationship matrix emphasizes the effect of causative variants with low frequencies and is therefore more successful in detecting these.

In terms of detecting variants, RHM performed least well when all the regional variation was generated by the effect of a single haplotype (1Hap) contrasting with all others in the region. In this scenario SNP alleles are not individually associated with the trait, unless they occur predominantly in the chosen haplotype, leading to the poor performance of RHM. RHM also failed to unbiasedly estimate the RH in this situation. Further investigation showed that RHM gave low LRT and also its significant LRT over-estimated the regional heritability when the frequency of the causative haplotype allele was low (<0.10) but otherwise performed similarly to HHM. When the causative allele is rare, SNP alleles will not match its frequency leading to poor performance of the SNP-based method. In general, haplotype alleles will have a frequency spectrum more similar to that expected from causal variants including many more rare variants than found in a SNP panel.

When the analysis window was substantially longer than that used to simulate the phenotypes, HHM gave lower LRT compared with analyzing using the correct window (see Supplementary Figure 2). This effect was less pronounced for RHM. Larger windows will increase the number of haplotypes in the window. A consequence is that some individuals that have identical haplotypes for the simulation are no longer identical in the analysis. Hence the estimated regional relationship is a less good model for the observed phenotypic similarities. For RHM the effect is less severe as the additional SNPs have less effect on the regional relationship matrix as the contribution from each SNP is additive.

Nagamine *et al*.^10^ used a SNP-based regional genomic relationship matrix where the analysis regions (windows) were defined using a fixed number of SNPs, ignoring the haplotype structure. Hence, the windows in their study may contain incomplete and/or several haplotype blocks. Therefore, in Nagamine *et al*.^10^ SNPs within a window that are used to construct the regional genomic relationship matrix may not all be in LD. Such windows also vary in their number and relative location between studies depending upon factors such as the genotyping array used, and SNPs omitted subsequent to quality control procedures. In the current study, we defined windows on the basis of the local haplotype structure, which is estimated as bounded by recombination hotspots, leading to windows containing only SNPs in LD with their neighbors and with different windows having different numbers of SNPs. Using recombination hotspots from a human reference panel to determine the haplotype windows has two advantages. First, the accuracy of determining windows using high-throughput sequence data from the reference data set will be higher than using the less-dense genotype information in the population. Second, the use of a reference panel to determine the windows ensures that the windows will be consistently defined over studies where different SNP-chips may be used. This allows comparisons across studies and the potential to combine results using a meta-analysis.

In this study we used two variance components based on the regional genomic relationship matrix and whole genome relationship matrix, as in the model proposed by Nagamine *et al*.^10^_._ When the data set contains related individuals, including the whole genome relationship matrix in the model will prevent overestimating the regional effect due to the potential similarity of the regional and whole genome relationships. Estimating two variance components is computationally demanding, so to speed up the genome scan, we recommend using one of the following two approximations. The simplest would be to analyse the data omitting the whole genome variance component, identifying the regions that most likely to harbour variants of interest and then repeating the analysis of these regions including the second variance component. The second obtains residuals fitting the whole genome relationship matrix and other explanatory variables, e.g. GRAMMAR + residuals^24^, to correct for the similarity between individuals. These residuals are subsequently used as the phenotype in an analysis fitting only the regional relationship matrix. This approach was shown to use 60% (165 seconds) less CPU time and 75% (4.8 gigabytes) less memory than the full model in a tested population (N = 7000). As we expect that LRT and RH may be affected differently by the approximations and thus we recommend to follow up the approximate analyses in regions of interest with analyses using the full two variance components.

In conclusion, our simulations suggest that our haplotype-based mapping (HHM) approach can capture a proportion of the missing heritability explained by rare haplotypes which are not detected by RHM and other SNP-based approaches. The HHM method detected rare variants along with common variants while the RHM approach was capable of detecting common variants. The HHM approach is a powerful tool for estimating RH and capturing rare variants through accurate estimation in all scenarios regardless of phenotype simulation, block and window size.

## Material and Methods

We propose the new method of Haplotype Heritability Mapping (HHM) which uses the genomic relationship matrix for a region estimated from haplotype information. These regional relationship coefficients can be used in a mixed model along with a second variance component which is used to explain the relationship between individuals caused by variation at loci elsewhere in the genome^25^. This whole genome genomic matrix in the haplotype-based approach is analogous to that used in RHM methods used in REACTA^26^ and GenABEL^27^. We investigate the performance of the HHM approach, using simulated data and compare with the RHM approach.

### SNP-based Regional Genomic Relationship Matrix

Using SNP genotypes the estimated relationship coefficient between individuals for a region of the genome can be estimated using equation 1^10,26,27^.

1 $$IB{S}_{ij}=\frac{1}{S}\sum _{k=1}^{S}\frac{({O}_{ik}-2{P}_{k})({O}_{jk}-2{P}_{k})}{2{P}_{k}(1-{P}_{k})}$$

where *IBS*_*ij*_ is the estimated genomic relationship between individual *i* and *j*; S is the number of SNPs in the region, *O*_*ik*_ and *O*_*jk*_ are the genotypes of the *i-th* and *j-th* individuals at the *k-th* SNP (coded as 0, 1, and 2 for AA, AB and BB, respectively); and *P*_*k*_ is the frequency of the counted allele (B in the example) at the *k-th* SNP.

### Haplotype-based Regional Genomic Relationship Matrix

The concept of regional relationship coefficients can be extended to consider haplotypes in the specified region rather than SNPs genotypes information. Analogous to the regional genomic relationship matrix in RHM, we propose a haplotype-based estimation as follows:

2 $$HIB{S}_{ij}=\frac{1}{H}\sum _{k=1}^{H}\frac{({Q}_{ik}-2{P}_{k})({Q}_{jk}-2{P}_{k})}{2{P}_{k}(1-{P}_{k})}$$

where *HIBS*_*ij*_ is the haplotype-based estimated genomic relationship between individual *i* and *j*; H is the number of haplotypes observed in the analysis window; *Q*_*ik*_ and *Q*_jk_ are the diplotypes of the *i-th* and *j-th* individuals for the *k-th* haplotype (each individual’s diplotype is recorded as the number of copies of this haplotype with 0 representing *D*_1_*D*_1_, 1 for *D*_1_*D*_*k*_/*D*_*k*_*D*_1_, and 2 for *D*_*k*_*D*_*k*_, when l not equal to k); and *P*_*k*_ is the frequency of *k-th* haplotype.

Previously the RHM method has been presented using a SNP-based relationship matrix^10^ For the HHM, we propose the new haplotype-based estimator can be used in an equivalent analysis. Following Nagamine *et al*.^10^ we use two random effects, one to account for the regional genetic variance and the other for the whole genome effect. In both cases the whole genome effect is modeled by the usual whole-genome SNP-based relationship matrix also known as the genomic relationship matrix.

We propose that the windows used should reflect the LD structure of the genome. Hence published recombination maps indicating recombination hotspots are used to delimit the windows. Thus, tightly linked adjacent SNPs will cluster in the same analysis window, using recombination hotspots as windows boundaries.

### Population and SNP array information for the simulation study

Samples were available from three Southern European cohorts: from the city of Split and islands of Vis and Korcula on the Dalmatian coast of Croatia. The study was approved by the Ethical Committee of the Medical School, University of Zagreb and the Multi-Centre Research Ethics Committee for Scotland. All participants gave written informed consent. The samples were genotyped using 300 K SNP genotyping arrays (Illumina Human Hap300 for Vis and Illumina CNV370 for Korcula and Split). Quality control procedures were performed per SNP and per individual. SNPs with minor allele frequency <0.01, out of Hardy-Weinberg equilibrium (P < 10^−8′^) and with a call rate <0.95 were discarded. Individuals with a call rate of <0.95 were excluded. After quality control, 2186 individuals and 267,136 autosomal SNPs genotyped in all the populations remained and were used in our analysis.

### Simulation

The haplotypes of the base population individuals were inferred from the available genotypes at 267136 SNPs of the 2186 individuals using BEAGLE (version 3.3.2)^12^. The base population individuals were randomly selected with replacement as 2186 pairs of parents of generation one in each 10 replicates of the simulation. The sex ratio was set to 1:1 and the fertility rate was one child per each mating. Subsequently, random mating was simulated for the next 20 generations. Population size was kept constant over generations at the base population size. In the current study, simulated genotypes from generation 20 were used.

Phenotypes were simulated with a total variance of one and heritability of 0.30, of which 0.25 was polygenic heritability for which all SNPs were assumed to have a very small effect on the phenotype and the remaining 0.05 was regional heritability (RH) contributed by a single region. Twenty different regions were selected for the regional component. Ten of these regions contained one of the top 10 SNPs reported in the meta-analysis of Teslovich *et al*.^28^ for HDL and the other 10 were control regions, each selected as the symmetrically placed SNP around the median SNP, considering SNP order, within the same chromosome as one of the reported hits. Haplotype blocks delimited by recombination hotspots with at least 5 centiMorgans per megabase (cM/Mb) based on the Genome Reference Consortium Human Build 37 (http://www.ncbi.nlm.nih.gov/projects/genome/assembly/grc/human/) were identified around each of the selected SNPs. The number of SNPs within the resulting blocks for this study ranged from 1 to 72.

Four scenarios were assumed to generate the regional genetic effect in each region, either using one SNP (1SNP) in the region, all SNPs (AllSNP) in the region (each one contributing equal variance), one randomly selected haplotype (1Hap) in the block or, finally, all haplotypes (AllHap) in the block having an effect. In the 1SNP scenario, in the meta-analysis of Teslovich *et al*.^28^ based regions, the causal SNP chosen in the simulation was the identified hit SNP from meta-analysis or the nearest SNP to it in our dataset and in the control regions, as considering SNP order on the chromosome, the SNP was the symmetrically placed SNP around the median SNP in the chromosome to the selected SNP in the meta-analysis based region. In the 1Hap scenario, one haplotype was selected randomly out of all available haplotypes in the block regardless of haplotype frequency. The summary of the haplotype frequencies for the haplotype selected to be causal in the 1Hap scenario is presented on Supplementary Table S1. The majority of the selected haplotypes in the 1Hap scenario had extremely low frequency (<0.020), therefore, the 1Hap scenario demonstrates a rare variant scenario. In the AllHap scenario, the effect of each haplotype on the simulated trait was randomly selected from a normal distribution and then the variance generated by the region was scaled to achieve the simulated regional heritability.

In total, for each of the four scenarios (1SNP, AllSNP, 1Hap, AllHap) there were 200 simulations representing the 10 replicates for each of the 20 regions. A summary of the simulated regional effect is presented in Supplementary Table S2.

### Mapping framework

The RHM was performed using two variance components, one based on the whole genome relationship matrix and the other based on the regional relationship matrix, as described by Nagamine *et al*.^10^. In the current study, haplotype windows were used as the regions for analysis, instead of using fixed-size windows containing a constant number of SNPs as in Nagamine *et al*.^10^. Two haplotype window sizes were considered for the analysis by determining haplotype boundaries through recombination hotspots with at least 5 cM/Mb or 10 cM/Mb in the Genome Reference Consortium Human Build 37. In the 5 cM/Mb boundary, the haplotype windows used in the analysis were identical to the causal blocks of the simulation, and in the 10 cM/Mb, the haplotype windows were either identical or larger than the simulated causal blocks. Descriptive statistic of 5 cM/Mb and 10 cM/Mb boundary are presented in Table 1. It should be noted that when the number of SNPs in a window is equal to one, the SNP-based and the haplotype-based regional genomic relationship matrices are identical.

**Table 1:** Window size in number of SNPs for the 5 cM/Mb and 10 cM/Mb recombination rate boundaries for each region used in the simulation study.

Region	5 cM/Mb				10 cM/Mb			
	Chr	Start	End	Size (NSNP)	Chr	Start	End	Size (NSNP)
A	2	19,615	19,615	1	2	19,615	19,615	1
B	16	2,786	2,786	1	16	2,786	2,786	1
C	16	4,025	4,025	1	16	4,025	4,027	3
D	11	7,263	7,264	2	11	7,239	7,264	26
E	9	8,298	8,301	4	9	8,298	8,317	20
F	9	9,793	9,796	4	9	9,793	9,797	5
G	15	3,293	3,296	4	15	3,293	3,297	5
H	8	12,690	12,694	5	8	12,675	12,697	23
I	8	3,059	3,065	7	8	3,057	3,065	9
J	11	2,268	2,275	8	11	2,268	2,275	8
K	11	5,470	5,477	8	11	5,437	5,485	49
L	16	3,790	3,798	9	16	3,790	3,798	9
M	11	10,464	10,473	10	11	10,464	10,473	10
N	15	4,413	4,424	12	15	4,413	4,424	12
O	2	2,441	2,461	21	2	2,441	2,465	25
P	8	1,503	1,523	21	8	1,503	1,547	45
Q	18	4,908	49,28	21	18	4,908	4,929	22
R	8	14,229	14,252	24	8	14,221	14,255	35
S	18	4,132	4,160	29	18	4,119	4,160	42
T	16	4,979	5,050	72	16	4,969	5,073	105

Chr: Chromosome; Start: Start SNP number; End: End SNP Number; Size: Window Size. NSNP: Number of SNPs in the window.

The whole genome relationship matrix and the regional genomic relationship matrix were constructed using custom-made scripts. The performance of each approach on detecting simulated variants was investigated by using REACTA version 0.9.7^26^ to solve the mixed model equations. Bonferroni corrected genome-wide 5% significance threshold were used to determine the level of significance, with the correction being based on the number of haplotype windows in the genome. For the 5 cM/Mb and 10 cM/Mb boundaries the genome was partitioned into 50,604 and 33,637 windows, respectively, resulting in Bonferroni corrected genome-wide likelihood ratio thresholds (LRT) of 21.60 for the 5 cM/Mb boundaries and 20.60 for the 10 cM/Mb.

### Ethics statement

All the Croatian cohorts received ethical approval from the Ethics Committee of the Medical School, University of Split and the NHS Lothian (South East Scotland Research Ethics Committee). The ORCADES study received ethical approval from the NHS Orkney Research Ethics Committee and North of Scotland Research Ethics Committee. All studies conformed to the ethical guidelines of the 1975 Declaration of Helsinki and were approved by appropriate ethics boards, with all participants signing informed consent prior to participation.

### Data availability

We have neither consent from individual participants nor appropriate ethical approval to permit full public release of data underlying this study. The datasets generated during and/or analysed during the current study are available from the first author on reasonable request.

## Electronic supplementary material

Supplementary materials
