# Supplementary material for: Haplotype Heritability Mapping Method Uncovers Missing Heritability of Complex Traits
Source: Sci Rep. 2018 Mar 21;8:4982. doi: 10.1038/s41598-018-23307-4 (PMC5862984; doi:10.1038/s41598-018-23307-4)

**Haplotype Heritability Mapping Method Uncovers Missing**

**Heritability of Complex Traits**

Masoud Shirali^1^, Sara A. Knott ^2^, Ricardo Pong-Wong ^3^, Pau Navarro ^1^, Chris S. Haley^1,3,*^

^1^ MRC Human Genetics Unit, MRC Institute of Genetics and Molecular Medicine, University of Edinburgh, Edinburgh, EH4 2XU, UK.

^2^ Institute of Evolutionary Biology, University of Edinburgh, Edinburgh, EH9 3FL, UK.

^3^ The Roslin Institute and R (D) SVS, University of Edinburgh, Easter Bush, Midlothian, EH25 9RG, UK.

Corresponding author: CSH (Chris.Haley@igmm.ed.ac.uk)

Tel: +44 (0) 131 651 8500

Fax: +44 (0) 131 651 8800

**Supplementary**

**Supplementary Table S1: Summary of haplotype frequency of the causal haplotype in the 1Hap simulation scenario.**

|  | **Region** | **Block Size** | **Mean** | **SD** | **Min** | **Max** |
| --- | --- | --- | --- | --- | --- | --- |
| A | 1 | 1 | 0.506 | 0.207 | 0.288 | 0.737 |
| B | 2 | 1 | 0.916 | 0.017 | 0.888 | 0.936 |
| C | 3 | 1 | 0.369 | 0.174 | 0.240 | 0.732 |
| D | 4 | 2 | 0.447 | 0.210 | 2.29 x 10^-4^ | 0.665 |
| E | 5 | 4 | 0.005 | 0.004 | 4.57 x 10^-4^ | 0.014 |
| F | 6 | 4 | 0.024 | 0.031 | 2.29 x 10^-4^ | 0.097 |
| G | 7 | 4 | 0.282 | 0.120 | 0.082 | 0.384 |
| H | 8 | 5 | 0.004 | 0.005 | 4.57 x 10^-4^ | 0.017 |
| I | 9 | 7 | 0.004 | 0.003 | 6.86 x 10^-4^ | 0.010 |
| J | 10 | 8 | 0.006 | 0.004 | 2.29 x 10^-4^ | 0.012 |
| K | 11 | 8 | 0.076 | 0.060 | 0.003 | 0.196 |
| L | 12 | 9 | 0.017 | 0.015 | 6.86 x 10^-4^ | 0.049 |
| M | 13 | 10 | 1.33 x 10^-3^ | 0.002 | 2.29 x 10^-4^ | 0.005 |
| N | 14 | 12 | 0.021 | 0.020 | 0.006 | 0.073 |
| O | 15 | 21 | 8.23 x 10^-4^ | 5.52 x 10^-4^ | 2.29 x 10^-4^ | 0.002 |
| P | 16 | 21 | 0.002 | 0.002 | 6.86 x10^-4^ | 0.006 |
| Q | 17 | 21 | 0.010 | 0.009 | 2.29 x 10^-4^ | 0.028 |
| R | 18 | 24 | 0.006 | 0.013 | 4.57 x 10^-4^ | 0.042 |
| S | 19 | 29 | 0.006 | 0.003 | 0.003 | 0.012 |
| T | 20 | 72 | 0.012 | 0.013 | 1.14 x 10^-3^ | 0.046 |

SD: Standard Deviation; Min: Minimum; Max: Maximum.

**Supplementary Table S2: Summary of simulated RH for 10 replicates of the 20 casual regions in the 1SNP, AllSNP, AllHap and 1Hap simulated scenario.**

| **Region** | **Block**  **size** | **1SNP** | | | |  | **AllSNP** | | | |  | **1Hap** | | | |  | | **AllHap** | | |
| --- | --- | --- | --- | --- | --- | --- | --- | --- | --- | --- | --- | --- | --- | --- | --- | --- | --- | --- | --- | --- |
|  |  | **Mean** | **SD** | **Min** | **Max** |  | **Mean** | **SD** | **Min** | **Max** |  | **Mean** | **SD** | **Min** | **Max** |  | **Mean** | **SD** | **Min** | **Max** |
| A | 1 | 0.049 | 0.001 | 0.047 | 0.051 |  | 0.049 | 0.001 | 0.047 | 0.051 |  | 0.050 | 0.001 | 0.049 | 0.052 |  | 0.050 | 0.001 | 0.049 | 0.051 |
| B | 1 | 0.049 | 0.002 | 0.047 | 0.051 |  | 0.049 | 0.001 | 0.047 | 0.051 |  | 0.050 | 0.001 | 0.049 | 0.051 |  | 0.050 | 0.001 | 0.049 | 0.052 |
| C | 1 | 0.049 | 0.001 | 0.048 | 0.051 |  | 0.049 | 0.001 | 0.048 | 0.050 |  | 0.050 | 0.001 | 0.049 | 0.052 |  | 0.050 | 0.001 | 0.048 | 0.051 |
| D | 2 | 0.049 | 0.001 | 0.047 | 0.051 |  | 0.049 | 0.001 | 0.047 | 0.051 |  | 0.050 | 0.001 | 0.048 | 0.052 |  | 0.050 | 0.001 | 0.048 | 0.051 |
| E | 4 | 0.049 | 0.001 | 0.047 | 0.051 |  | 0.049 | 0.001 | 0.047 | 0.051 |  | 0.050 | 0.001 | 0.048 | 0.051 |  | 0.050 | 0.001 | 0.048 | 0.052 |
| F | 4 | 0.049 | 0.001 | 0.048 | 0.051 |  | 0.049 | 0.001 | 0.048 | 0.050 |  | 0.050 | 0.001 | 0.049 | 0.052 |  | 0.050 | 0.001 | 0.048 | 0.051 |
| G | 4 | 0.049 | 0.001 | 0.048 | 0.050 |  | 0.049 | 0.001 | 0.047 | 0.051 |  | 0.050 | 0.001 | 0.049 | 0.051 |  | 0.050 | 0.001 | 0.049 | 0.052 |
| H | 5 | 0.049 | 0.001 | 0.048 | 0.051 |  | 0.049 | 0.001 | 0.047 | 0.050 |  | 0.050 | 0.001 | 0.049 | 0.052 |  | 0.050 | 0.001 | 0.048 | 0.051 |
| I | 7 | 0.049 | 0.001 | 0.047 | 0.051 |  | 0.049 | 0.001 | 0.047 | 0.051 |  | 0.050 | 0.001 | 0.049 | 0.052 |  | 0.050 | 0.001 | 0.048 | 0.052 |
| J | 8 | 0.049 | 0.001 | 0.048 | 0.051 |  | 0.049 | 0.001 | 0.048 | 0.051 |  | 0.050 | 0.001 | 0.049 | 0.051 |  | 0.050 | 0.001 | 0.049 | 0.052 |
| K | 8 | 0.049 | 0.001 | 0.047 | 0.051 |  | 0.049 | 0.001 | 0.048 | 0.050 |  | 0.050 | 0.000 | 0.049 | 0.051 |  | 0.050 | 0.001 | 0.049 | 0.052 |
| L | 9 | 0.049 | 0.001 | 0.047 | 0.050 |  | 0.049 | 0.001 | 0.047 | 0.051 |  | 0.050 | 0.001 | 0.049 | 0.051 |  | 0.050 | 0.001 | 0.049 | 0.051 |
| M | 10 | 0.049 | 0.001 | 0.047 | 0.050 |  | 0.049 | 0.001 | 0.048 | 0.050 |  | 0.050 | 0.001 | 0.048 | 0.051 |  | 0.050 | 0.001 | 0.048 | 0.051 |
| N | 12 | 0.049 | 0.001 | 0.047 | 0.050 |  | 0.049 | 0.001 | 0.047 | 0.051 |  | 0.050 | 0.001 | 0.048 | 0.052 |  | 0.050 | 0.001 | 0.049 | 0.052 |
| O | 21 | 0.049 | 0.001 | 0.048 | 0.051 |  | 0.049 | 0.001 | 0.047 | 0.051 |  | 0.050 | 0.001 | 0.048 | 0.051 |  | 0.050 | 0.001 | 0.049 | 0.051 |
| P | 21 | 0.049 | 0.001 | 0.047 | 0.050 |  | 0.049 | 0.001 | 0.047 | 0.050 |  | 0.050 | 0.001 | 0.049 | 0.051 |  | 0.050 | 0.001 | 0.049 | 0.051 |
| Q | 21 | 0.049 | 0.001 | 0.048 | 0.051 |  | 0.049 | 0.001 | 0.048 | 0.051 |  | 0.050 | 0.001 | 0.049 | 0.051 |  | 0.050 | 0.001 | 0.046 | 0.051 |
| R | 24 | 0.049 | 0.001 | 0.047 | 0.050 |  | 0.049 | 0.001 | 0.047 | 0.050 |  | 0.050 | 0.001 | 0.048 | 0.052 |  | 0.050 | 0.001 | 0.049 | 0.053 |
| S | 29 | 0.049 | 0.001 | 0.048 | 0.051 |  | 0.049 | 0.001 | 0.047 | 0.050 |  | 0.050 | 0.001 | 0.049 | 0.051 |  | 0.050 | 0.001 | 0.048 | 0.052 |
| T | 72 | 0.049 | 0.001 | 0.047 | 0.051 |  | 0.005 | 0.015 | 0.000 | 0.049 |  | 0.050 | 0.001 | 0.049 | 0.052 |  | 0.050 | 0.001 | 0.048 | 0.051 |

RH: Regional Heritability; SD: Standard Deviation; Min: Minimum; Max: Maximum.

**Supplementary Table S3: Estimated LRT for the 1SNP simulated scenario for the 20 regions obtained using either the Haplotype Heritability Mapping method (HHM) or the Regional Heritability Mapping method (RHM) in 5cM/Mb boundary.**

|  | **Window**  **size** |  | **HHM** | | | |  | **RHM** | | | |
| --- | --- | --- | --- | --- | --- | --- | --- | --- | --- | --- | --- |
| **Region** |  |  | **Mean** | **SD** | **Min** | **Max** |  | **Mean** | **SD** | **Min** | **Max** |
| A | 1 |  | 94.60 | 16.27 | 71.91 | 120.46 |  | 94.60 | 16.27 | 71.91 | 120.47 |
| B | 1 |  | 105.59 | 23.47 | 75.41 | 137.06 |  | 105.59 | 23.47 | 75.41 | 137.06 |
| C | 1 |  | 95.34 | 21.57 | 63.43 | 124.18 |  | 95.33 | 21.56 | 63.44 | 124.17 |
| D | 2 |  | 104.07 | 23.05 | 60.03 | 134.04 |  | 103.75 | 23.01 | 60.02 | 134.07 |
| E | 4 |  | 84.05 | 17.41 | 65.84 | 115.04 |  | 90.25 | 16.96 | 76.70 | 123.57 |
| F | 4 |  | 75.63 | 21.09 | 51.24 | 107.90 |  | 80.60 | 20.61 | 56.30 | 110.04 |
| G | 4 |  | 87.43 | 12.71 | 67.62 | 107.75 |  | 92.44 | 13.42 | 73.68 | 116.52 |
| H | 5 |  | 81.45 | 18.07 | 53.60 | 114.40 |  | 96.14 | 17.12 | 73.43 | 126.84 |
| I | 7 |  | 72.39 | 25.21 | 44.37 | 122.23 |  | 84.86 | 26.05 | 61.64 | 140.44 |
| J | 8 |  | 58.17 | 14.82 | 30.50 | 71.81 |  | 76.76 | 15.80 | 43.93 | 92.33 |
| K | 8 |  | 64.69 | 20.24 | 29.29 | 90.17 |  | 79.49 | 21.92 | 41.19 | 107.91 |
| L | 9 |  | 60.09 | 10.54 | 43.34 | 79.39 |  | 80.58 | 10.12 | 64.22 | 97.32 |
| M | 10 |  | 60.60 | 20.99 | 28.10 | 88.85 |  | 82.88 | 23.35 | 48.46 | 117.28 |
| N | 12 |  | 50.19 | 17.32 | 30.57 | 94.85 |  | 83.69 | 19.85 | 63.32 | 131.57 |
| O | 21 |  | 27.83 | 13.22 | 2.06 | 52.23 |  | 68.36 | 15.22 | 36.40 | 87.65 |
| P | 21 |  | 39.10 | 12.34 | 26.29 | 70.21 |  | 82.47 | 13.94 | 64.65 | 114.43 |
| Q | 21 |  | 56.61 | 20.33 | 32.32 | 105.32 |  | 90.73 | 25.43 | 63.17 | 147.18 |
| R | 24 |  | 27.40 | 9.97 | 14.73 | 47.54 |  | 66.51 | 14.31 | 54.12 | 94.56 |
| S | 29 |  | 35.65 | 12.74 | 14.93 | 57.82 |  | 83.56 | 19.65 | 50.32 | 120.88 |
| T | 72 |  | 18.63 | 11.09 | 6.10 | 40.70 |  | 70.06 | 18.64 | 38.48 | 100.98 |

LRT: Likelihood Ratio Test; SD: Standard Deviation; Min: Minimum; Max: Maximum; The RHM and HMM estimates for 1 SNP windows are very similar.

**Supplementary Table S4: Estimated RH for the 1SNP simulated scenario for the 20 regions obtained using either the Haplotype Heritability Mapping method (HHM) or the Regional Heritability Mapping method (RHM) in 5cM/Mb boundary.**

|  | **Window**  **Size** |  | **HHM** | | | |  | **RHM** | | | |
| --- | --- | --- | --- | --- | --- | --- | --- | --- | --- | --- | --- |
| **Region** |  |  | **Mean** | **SD** | **Min** | **Max** |  | **Mean** | **SD** | **Min** | **Max** |
| A | 1 |  | 0.048 | 0.007 | 0.037 | 0.060 |  | 0.048 | 0.008 | 0.037 | 0.060 |
| B | 1 |  | 0.053 | 0.012 | 0.038 | 0.069 |  | 0.053 | 0.012 | 0.038 | 0.068 |
| C | 1 |  | 0.048 | 0.010 | 0.033 | 0.062 |  | 0.048 | 0.010 | 0.033 | 0.062 |
| D | 2 |  | 0.051 | 0.011 | 0.031 | 0.067 |  | 0.106 | 0.169 | 0.031 | 0.585 |
| E | 4 |  | 0.045 | 0.008 | 0.037 | 0.061 |  | 0.056 | 0.021 | 0.034 | 0.096 |
| F | 4 |  | 0.041 | 0.009 | 0.029 | 0.055 |  | 0.037 | 0.010 | 0.022 | 0.057 |
| G | 4 |  | 0.041 | 0.006 | 0.032 | 0.051 |  | 0.056 | 0.044 | 0.025 | 0.171 |
| H | 5 |  | 0.044 | 0.007 | 0.031 | 0.056 |  | 0.048 | 0.007 | 0.035 | 0.057 |
| I | 7 |  | 0.044 | 0.012 | 0.032 | 0.068 |  | 0.047 | 0.028 | 0.026 | 0.120 |
| J | 8 |  | 0.043 | 0.008 | 0.026 | 0.050 |  | 0.045 | 0.012 | 0.027 | 0.060 |
| K | 8 |  | 0.046 | 0.011 | 0.025 | 0.059 |  | 0.057 | 0.017 | 0.032 | 0.081 |
| L | 9 |  | 0.042 | 0.006 | 0.032 | 0.052 |  | 0.063 | 0.020 | 0.037 | 0.088 |
| M | 10 |  | 0.045 | 0.011 | 0.026 | 0.059 |  | 0.072 | 0.017 | 0.044 | 0.095 |
| N | 12 |  | 0.049 | 0.011 | 0.036 | 0.079 |  | 0.040 | 0.011 | 0.028 | 0.066 |
| O | 21 |  | 0.046 | 0.015 | 0.012 | 0.070 |  | 0.057 | 0.020 | 0.027 | 0.100 |
| P | 21 |  | 0.046 | 0.009 | 0.034 | 0.070 |  | 0.054 | 0.011 | 0.041 | 0.073 |
| Q | 21 |  | 0.052 | 0.013 | 0.035 | 0.079 |  | 0.063 | 0.032 | 0.024 | 0.139 |
| R | 24 |  | 0.042 | 0.009 | 0.031 | 0.062 |  | 0.054 | 0.012 | 0.040 | 0.073 |
| S | 29 |  | 0.050 | 0.011 | 0.031 | 0.069 |  | 0.045 | 0.012 | 0.030 | 0.062 |
| T | 72 |  | 0.048 | 0.017 | 0.027 | 0.078 |  | 0.050 | 0.015 | 0.024 | 0.072 |

RH: Regional Heritability; SD: Standard Deviation; Min: Minimum; Max: Maximum; The RHM and HMM estimates for 1 SNP windows are very similar.

**Supplementary Table S5: Estimated LRT for the AllSNP simulated scenario for the 20 regions obtained using either the Haplotype Heritability Mapping method (HHM) or the Regional Heritability Mapping method (RHM) in 5cM/Mb boundary.**

|  | **Window**  **size** |  | **HHM** | | | |  | **RHM** | | | |
| --- | --- | --- | --- | --- | --- | --- | --- | --- | --- | --- | --- |
| **Region** |  |  | **Mean** | **SD** | **Min** | **Max** |  | **Mean** | **SD** | **Min** | **Max** |
| A | 1 |  | 94.60 | 16.27 | 71.91 | 120.46 |  | 94.60 | 16.27 | 71.91 | 120.47 |
| B | 1 |  | 105.59 | 23.47 | 75.41 | 137.06 |  | 105.59 | 23.47 | 75.41 | 137.06 |
| C | 1 |  | 95.34 | 21.57 | 63.43 | 124.18 |  | 95.33 | 21.56 | 63.44 | 124.17 |
| D | 2 |  | 16.82 | 52.13 | 0.00 | 165.19 |  | 16.02 | 49.60 | 0.00 | 157.18 |
| E | 4 |  | 87.10 | 21.29 | 58.42 | 127.44 |  | 92.41 | 22.47 | 66.19 | 136.90 |
| F | 4 |  | 81.69 | 15.64 | 48.46 | 104.08 |  | 85.48 | 15.50 | 52.23 | 108.11 |
| G | 4 |  | 84.06 | 18.13 | 58.68 | 114.75 |  | 90.04 | 19.06 | 66.66 | 124.04 |
| H | 5 |  | 80.98 | 15.45 | 61.91 | 101.93 |  | 95.50 | 15.55 | 75.56 | 116.34 |
| I | 7 |  | 72.18 | 21.05 | 41.60 | 104.87 |  | 80.23 | 21.52 | 53.94 | 120.41 |
| J | 8 |  | 61.53 | 20.05 | 24.45 | 84.44 |  | 77.59 | 22.35 | 37.15 | 100.78 |
| K | 8 |  | 65.21 | 18.84 | 31.76 | 96.56 |  | 78.44 | 20.07 | 42.75 | 107.80 |
| L | 9 |  | 60.51 | 15.82 | 37.89 | 92.59 |  | 77.65 | 18.24 | 55.16 | 111.79 |
| M | 10 |  | 64.22 | 18.77 | 34.83 | 87.47 |  | 89.73 | 20.99 | 56.36 | 119.59 |
| N | 12 |  | 49.02 | 10.35 | 31.21 | 63.66 |  | 78.50 | 12.87 | 56.42 | 98.93 |
| O | 21 |  | 35.03 | 19.57 | 8.81 | 78.53 |  | 75.70 | 23.34 | 35.61 | 126.64 |
| P | 21 |  | 37.29 | 11.48 | 25.62 | 56.39 |  | 78.54 | 15.86 | 60.24 | 109.08 |
| Q | 21 |  | 53.76 | 11.78 | 43.25 | 74.44 |  | 84.72 | 16.23 | 69.99 | 117.72 |
| R | 24 |  | 34.93 | 9.61 | 21.40 | 53.96 |  | 72.86 | 12.53 | 54.59 | 91.44 |
| S | 29 |  | 32.93 | 8.68 | 15.13 | 47.60 |  | 77.26 | 8.69 | 61.22 | 91.08 |
| T | 72 |  | 20.63 | 12.25 | 1.35 | 34.54 |  | 72.28 | 22.49 | 36.54 | 94.77 |

LRT: Likelihood Ratio Test; SD: Standard Deviation; Min: Minimum; Max: Maximum; The RHM and HMM estimates for 1 SNP windows are very similar.

**Supplementary Table S6: Estimated RH for the AllSNP simulated scenario for the 20 regions obtained using either the Haplotype Heritability Mapping method (HHM) or the Regional Heritability Mapping method (RHM) in 5cM/Mb boundary.**

|  | **Window**  **size** |  | **HHM** | | | |  | **RHM** | | | |
| --- | --- | --- | --- | --- | --- | --- | --- | --- | --- | --- | --- |
| **Region** |  |  | **Mean** | **SD** | **Min** | **Max** |  | **Mean** | **SD** | **Min** | **Max** |
| A | 1 |  | 0.048 | 0.007 | 0.037 | 0.060 |  | 0.048 | 0.008 | 0.037 | 0.060 |
| B | 1 |  | 0.053 | 0.012 | 0.038 | 0.069 |  | 0.053 | 0.012 | 0.038 | 0.068 |
| C | 1 |  | 0.048 | 0.010 | 0.033 | 0.062 |  | 0.048 | 0.010 | 0.033 | 0.062 |
| D | 2 |  | 0.011 | 0.034 | 0.000 | 0.108 |  | 0.100 | 0.314 | 0.000 | 0.993 |
| E | 4 |  | 0.051 | 0.011 | 0.036 | 0.070 |  | 0.089 | 0.030 | 0.051 | 0.129 |
| F | 4 |  | 0.047 | 0.008 | 0.030 | 0.057 |  | 0.070 | 0.013 | 0.051 | 0.087 |
| G | 4 |  | 0.042 | 0.008 | 0.030 | 0.054 |  | 0.042 | 0.016 | 0.029 | 0.083 |
| H | 5 |  | 0.045 | 0.006 | 0.037 | 0.053 |  | 0.057 | 0.009 | 0.042 | 0.072 |
| I | 7 |  | 0.048 | 0.010 | 0.032 | 0.063 |  | 0.120 | 0.048 | 0.061 | 0.218 |
| J | 8 |  | 0.044 | 0.011 | 0.023 | 0.055 |  | 0.076 | 0.026 | 0.036 | 0.113 |
| K | 8 |  | 0.040 | 0.010 | 0.022 | 0.055 |  | 0.047 | 0.012 | 0.023 | 0.063 |
| L | 9 |  | 0.045 | 0.009 | 0.033 | 0.063 |  | 0.122 | 0.033 | 0.081 | 0.190 |
| M | 10 |  | 0.043 | 0.009 | 0.027 | 0.054 |  | 0.037 | 0.008 | 0.024 | 0.045 |
| N | 12 |  | 0.049 | 0.007 | 0.034 | 0.058 |  | 0.060 | 0.016 | 0.035 | 0.087 |
| O | 21 |  | 0.053 | 0.018 | 0.024 | 0.089 |  | 0.084 | 0.027 | 0.038 | 0.135 |
| P | 21 |  | 0.045 | 0.008 | 0.037 | 0.061 |  | 0.061 | 0.011 | 0.049 | 0.084 |
| Q | 21 |  | 0.050 | 0.008 | 0.041 | 0.064 |  | 0.066 | 0.021 | 0.040 | 0.118 |
| R | 24 |  | 0.047 | 0.006 | 0.036 | 0.057 |  | 0.060 | 0.021 | 0.038 | 0.101 |
| S | 29 |  | 0.048 | 0.007 | 0.031 | 0.059 |  | 0.052 | 0.012 | 0.027 | 0.066 |
| T | 72 |  | 0.050 | 0.021 | 0.012 | 0.072 |  | 0.063 | 0.022 | 0.026 | 0.090 |

RH: Regional Heritability; SD: Standard Deviation; Min: Minimum; Max: Maximum; The RHM and HMM estimates for 1 SNP windows are very similar.

**Supplementary Table S7: Estimated LRT for the 1Hap simulated scenario for the 20 regions obtained using either the Haplotype Heritability Mapping method (HHM) or the Regional Heritability Mapping method (RHM) in 5cM/Mb boundary.**

|  | **Window**  **Size** |  | **HHM** | | | |  | **RHM** | | | |
| --- | --- | --- | --- | --- | --- | --- | --- | --- | --- | --- | --- |
| **Region** |  |  | **Mean** | **SD** | **Min** | **Max** |  | **Mean** | **SD** | **Min** | **Max** |
| A | 1 |  | 106.16 | 17.12 | 88.31 | 141.64 |  | 106.17 | 17.13 | 88.32 | 141.64 |
| B | 1 |  | 89.72 | 18.56 | 60.92 | 119.30 |  | 89.72 | 18.56 | 60.91 | 119.30 |
| C | 1 |  | 94.73 | 19.78 | 69.92 | 139.02 |  | 94.73 | 19.77 | 69.91 | 139.00 |
| D | 2 |  | 101.35 | 20.95 | 73.20 | 135.17 |  | 97.56 | 28.62 | 35.33 | 135.17 |
| E | 4 |  | 85.87 | 16.79 | 65.02 | 126.25 |  | 4.89 | 11.82 | 0.00 | 38.08 |
| F | 4 |  | 93.59 | 15.21 | 64.66 | 110.20 |  | 40.30 | 30.59 | 0.00 | 90.35 |
| G | 4 |  | 92.49 | 10.22 | 81.34 | 107.70 |  | 94.64 | 10.85 | 76.67 | 110.47 |
| H | 5 |  | 85.05 | 23.85 | 40.88 | 136.48 |  | 5.10 | 9.56 | 0 | 30.16 |
| I | 7 |  | 81.03 | 22.00 | 54.78 | 127.17 |  | 8.33 | 8.59 | 0 | 24.58 |
| J | 8 |  | 74.63 | 25.18 | 42.37 | 123.23 |  | 8.55 | 12.99 | 0.00 | 33.67 |
| K | 8 |  | 86.28 | 15.54 | 66.56 | 110.47 |  | 72.93 | 35.92 | 2.81 | 118.31 |
| L | 9 |  | 62.89 | 16.29 | 37.59 | 98.55 |  | 29.46 | 29.27 | 0.00 | 79.14 |
| M | 10 |  | 65.25 | 14.82 | 41.83 | 89.58 |  | 0.79 | 0.91 | 0.00 | 2.63 |
| N | 12 |  | 56.91 | 20.49 | 37.43 | 100.02 |  | 14.64 | 19.67 | 0.00 | 64.01 |
| O | 21 |  | 38.60 | 15.38 | 18.96 | 65.22 |  | 0.82 | 1.60 | 0.00 | 5.23 |
| P | 21 |  | 36.20 | 13.76 | 14.67 | 51.04 |  | 1.55 | 3.12 | 0.00 | 10.16 |
| Q | 21 |  | 47.12 | 12.61 | 28.88 | 64.70 |  | 17.93 | 25.05 | 0.00 | 67.47 |
| R | 24 |  | 39.85 | 13.59 | 17.50 | 60.80 |  | 1.03 | 1.27 | 0.00 | 2.86 |
| S | 29 |  | 35.15 | 12.94 | 18.16 | 58.57 |  | 4.27 | 4.50 | 0.00 | 11.34 |
| T | 72 |  | 19.44 | 7.89 | 8.92 | 34.59 |  | 7.88 | 10.57 | 0.00 | 29.67 |

LRT: Likelihood Ratio Test; SD: Standard Deviation; Min: Minimum; Max: Maximum; The RHM and HMM estimates for 1 SNP windows are very similar.

**Supplementary Table S8: Estimated RH for the 1Hap simulated scenario for the 20 regions obtained using either the Haplotype Heritability Mapping method (HHM) or the Regional Heritability Mapping method (RHM) in 5cM/Mb boundary.**

|  | **Window**  **size** |  | **HHM** | | | |  | **RHM** | | | |
| --- | --- | --- | --- | --- | --- | --- | --- | --- | --- | --- | --- |
| **Region** |  |  | **Mean** | **SD** | **Min** | **Max** |  | **Mean** | **SD** | **Min** | **Max** |
| A | 1 |  | 0.053 | 0.007 | 0.046 | 0.069 |  | 0.054 | 0.008 | 0.045 | 0.069 |
| B | 1 |  | 0.045 | 0.008 | 0.033 | 0.058 |  | 0.045 | 0.008 | 0.033 | 0.058 |
| C | 1 |  | 0.048 | 0.010 | 0.036 | 0.069 |  | 0.048 | 0.010 | 0.036 | 0.068 |
| D | 2 |  | 0.052 | 0.009 | 0.040 | 0.067 |  | 0.116 | 0.202 | 0.040 | 0.689 |
| E | 4 |  | 0.054 | 0.008 | 0.043 | 0.073 |  | 0.017 | 0.043 | 0.000 | 0.137 |
| F | 4 |  | 0.058 | 0.007 | 0.043 | 0.065 |  | 0.273 | 0.296 | 0.000 | 0.739 |
| G | 4 |  | 0.045 | 0.004 | 0.040 | 0.052 |  | 0.066 | 0.024 | 0.031 | 0.112 |
| H | 5 |  | 0.057 | 0.013 | 0.032 | 0.083 |  | 0.008 | 0.011 | 0.000 | 0.031 |
| I | 7 |  | 0.055 | 0.011 | 0.044 | 0.078 |  | 0.135 | 0.159 | 0.000 | 0.441 |
| J | 8 |  | 0.055 | 0.013 | 0.037 | 0.080 |  | 0.070 | 0.181 | 0.000 | 0.582 |
| K | 8 |  | 0.055 | 0.008 | 0.044 | 0.073 |  | 0.084 | 0.081 | 0.010 | 0.306 |
| L | 9 |  | 0.048 | 0.009 | 0.033 | 0.067 |  | 0.141 | 0.175 | 0.000 | 0.473 |
| M | 10 |  | 0.050 | 0.008 | 0.038 | 0.062 |  | 0.015 | 0.043 | 0.000 | 0.138 |
| N | 12 |  | 0.056 | 0.013 | 0.044 | 0.084 |  | 0.034 | 0.032 | 0.000 | 0.085 |
| O | 21 |  | 0.055 | 0.013 | 0.037 | 0.077 |  | 0.003 | 0.004 | 0.000 | 0.011 |
| P | 21 |  | 0.044 | 0.010 | 0.027 | 0.055 |  | 0.004 | 0.007 | 0.000 | 0.023 |
| Q | 21 |  | 0.047 | 0.008 | 0.034 | 0.062 |  | 0.063 | 0.080 | 0.000 | 0.207 |
| R | 24 |  | 0.052 | 0.011 | 0.032 | 0.069 |  | 0.002 | 0.003 | 0.000 | 0.007 |
| S | 29 |  | 0.051 | 0.010 | 0.035 | 0.069 |  | 0.022 | 0.028 | 0.000 | 0.085 |
| T | 72 |  | 0.049 | 0.011 | 0.033 | 0.069 |  | 0.026 | 0.037 | 0.000 | 0.108 |

RH: Regional Heritability; SD: Standard Deviation; Min: Minimum; Max: Maximum; The RHM and HMM estimates for 1 SNP windows are very similar.

**Supplementary Table S9: Estimated LRT for the AllHap simulated scenario for the 20 regions obtained using either the Haplotype Heritability Mapping method (HHM) or the Regional Heritability Mapping method (RHM) in 5cM/Mb boundary.**

|  | **Window**  **size** |  | **HHM** | | | |  | **RHM** | | | |
| --- | --- | --- | --- | --- | --- | --- | --- | --- | --- | --- | --- |
| **Region** |  |  | **Mean** | **SD** | **Min** | **Max** |  | **Mean** | **SD** | **Min** | **Max** |
| A | 1 |  | 99.26 | 16.78 | 81.46 | 128.95 |  | 99.26 | 16.78 | 81.46 | 128.93 |
| B | 1 |  | 101.30 | 27.68 | 48.66 | 130.94 |  | 101.30 | 27.68 | 48.66 | 130.94 |
| C | 1 |  | 96.93 | 22.74 | 63.96 | 142.64 |  | 96.93 | 22.75 | 63.96 | 142.65 |
| D | 2 |  | 104.07 | 13.23 | 84.67 | 124.21 |  | 104.32 | 13.32 | 84.67 | 124.21 |
| E | 4 |  | 93.01 | 18.32 | 69.39 | 122.99 |  | 79.60 | 29.61 | 29.32 | 134.60 |
| F | 4 |  | 96.15 | 8.74 | 84.03 | 110.84 |  | 85.69 | 14.93 | 53.42 | 104.22 |
| G | 4 |  | 87.40 | 15.56 | 58.59 | 111.05 |  | 85.01 | 10.33 | 64.85 | 98.16 |
| H | 5 |  | 77.93 | 15.78 | 59.22 | 107.12 |  | 66.17 | 27.05 | 21.13 | 108.71 |
| I | 7 |  | 79.78 | 14.27 | 52.91 | 103.46 |  | 88.24 | 17.23 | 63.07 | 115.62 |
| J | 8 |  | 56.34 | 14.48 | 36.25 | 83.24 |  | 55.77 | 22.01 | 22.13 | 106.80 |
| K | 8 |  | 73.20 | 23.84 | 43.66 | 115.72 |  | 68.81 | 27.98 | 27.07 | 113.13 |
| L | 9 |  | 76.87 | 20.21 | 52.65 | 111.45 |  | 55.86 | 14.53 | 35.68 | 82.01 |
| M | 10 |  | 58.48 | 12.10 | 33.31 | 73.69 |  | 53.43 | 20.39 | 30.04 | 83.87 |
| N | 12 |  | 48.44 | 14.99 | 26.36 | 75.23 |  | 51.12 | 19.12 | 27.38 | 84.92 |
| O | 21 |  | 33.74 | 12.77 | 19.89 | 59.31 |  | 21.39 | 15.02 | 1.63 | 39.72 |
| P | 21 |  | 43.82 | 15.94 | 26.43 | 75.14 |  | 33.47 | 17.21 | 5.49 | 66.50 |
| Q | 21 |  | 45.74 | 23.30 | 29.44 | 107.52 |  | 52.98 | 26.18 | 20.52 | 116.36 |
| R | 24 |  | 39.13 | 24.54 | 11.26 | 92.98 |  | 24.96 | 25.52 | 5.71 | 91.40 |
| S | 29 |  | 29.98 | 6.95 | 21.26 | 43.24 |  | 20.10 | 8.64 | 3.59 | 29.74 |
| T | 72 |  | 18.41 | 8.85 | 6.34 | 30.73 |  | 8.54 | 9.47 | 0.00 | 28.40 |

LRT: Likelihood Ratio Test; SD: Standard Deviation; Min: Minimum; Max: Maximum; The RHM and HMM estimates for 1 SNP windows are very similar.

**Supplementary Table S10: Estimated RH for the AllHap simulated scenario for the 20 regions obtained using either the Haplotype Heritability Mapping method (HHM) or the Regional Heritability Mapping method (RHM) in 5cM/Mb boundary.**

|  | **Window**  **size** |  | **HHM** | | | |  | **RHM** | | | |
| --- | --- | --- | --- | --- | --- | --- | --- | --- | --- | --- | --- |
| **Region** |  |  | **Mean** | **SD** | **Min** | **Max** |  | **Mean** | **SD** | **Min** | **Max** |
| A | 1 |  | 0.050 | 0.008 | 0.043 | 0.065 |  | 0.050 | 0.008 | 0.043 | 0.065 |
| B | 1 |  | 0.051 | 0.013 | 0.027 | 0.063 |  | 0.051 | 0.013 | 0.027 | 0.063 |
| C | 1 |  | 0.049 | 0.011 | 0.032 | 0.071 |  | 0.049 | 0.012 | 0.032 | 0.071 |
| D | 2 |  | 0.051 | 0.007 | 0.043 | 0.062 |  | 0.052 | 0.006 | 0.043 | 0.062 |
| E | 4 |  | 0.049 | 0.008 | 0.035 | 0.063 |  | 0.072 | 0.054 | 0.028 | 0.180 |
| F | 4 |  | 0.050 | 0.004 | 0.042 | 0.055 |  | 0.051 | 0.021 | 0.028 | 0.102 |
| G | 4 |  | 0.045 | 0.009 | 0.035 | 0.062 |  | 0.114 | 0.158 | 0.024 | 0.548 |
| H | 5 |  | 0.046 | 0.006 | 0.038 | 0.055 |  | 0.041 | 0.021 | 0.015 | 0.091 |
| I | 7 |  | 0.047 | 0.007 | 0.034 | 0.059 |  | 0.081 | 0.056 | 0.031 | 0.189 |
| J | 8 |  | 0.041 | 0.007 | 0.033 | 0.051 |  | 0.076 | 0.082 | 0.033 | 0.303 |
| K | 8 |  | 0.044 | 0.010 | 0.034 | 0.067 |  | 0.048 | 0.013 | 0.020 | 0.061 |
| L | 9 |  | 0.052 | 0.013 | 0.037 | 0.075 |  | 0.100 | 0.089 | 0.026 | 0.331 |
| M | 10 |  | 0.044 | 0.008 | 0.027 | 0.052 |  | 0.042 | 0.021 | 0.015 | 0.090 |
| N | 12 |  | 0.047 | 0.010 | 0.032 | 0.066 |  | 0.046 | 0.017 | 0.023 | 0.070 |
| O | 21 |  | 0.053 | 0.012 | 0.038 | 0.076 |  | 0.030 | 0.022 | 0.005 | 0.071 |
| P | 21 |  | 0.049 | 0.011 | 0.037 | 0.070 |  | 0.045 | 0.033 | 0.017 | 0.120 |
| Q | 21 |  | 0.044 | 0.015 | 0.034 | 0.082 |  | 0.059 | 0.027 | 0.031 | 0.111 |
| R | 24 |  | 0.050 | 0.019 | 0.025 | 0.085 |  | 0.026 | 0.015 | 0.005 | 0.049 |
| S | 29 |  | 0.046 | 0.007 | 0.038 | 0.057 |  | 0.044 | 0.032 | 0.013 | 0.109 |
| T | 72 |  | 0.047 | 0.013 | 0.028 | 0.064 |  | 0.018 | 0.018 | 0.000 | 0.054 |

RH: Regional Heritability; SD: Standard Deviation; Min: Minimum; Max: Maximum; The RHM and HMM estimates for 1 SNP windows are very similar.

**Supplementary Figure S1: Average LRT (A,C,E,G) and RH (B,D,F,H) for the 20 window regions ordered by size in the 1SNP, AllSNP, AllHap, 1Hap simulations in 5cM/Mb boundary.** y-axis: in a, LRT(likelihood ratio test) is a statistical test to compare the goodness of fit of regional genomic relationship matrix in the model with the null model, and the blue line represent the 5% significance level Bonferroni corrected threshold. In b, RH (regional heritability) is proportion of trait variance explained by the regional genomic relationship matrix. X-axis: Number of SNPs in the window represent the size of analyzed region which is the same as the size of simulated region. The error bar represents the standard error of mean.


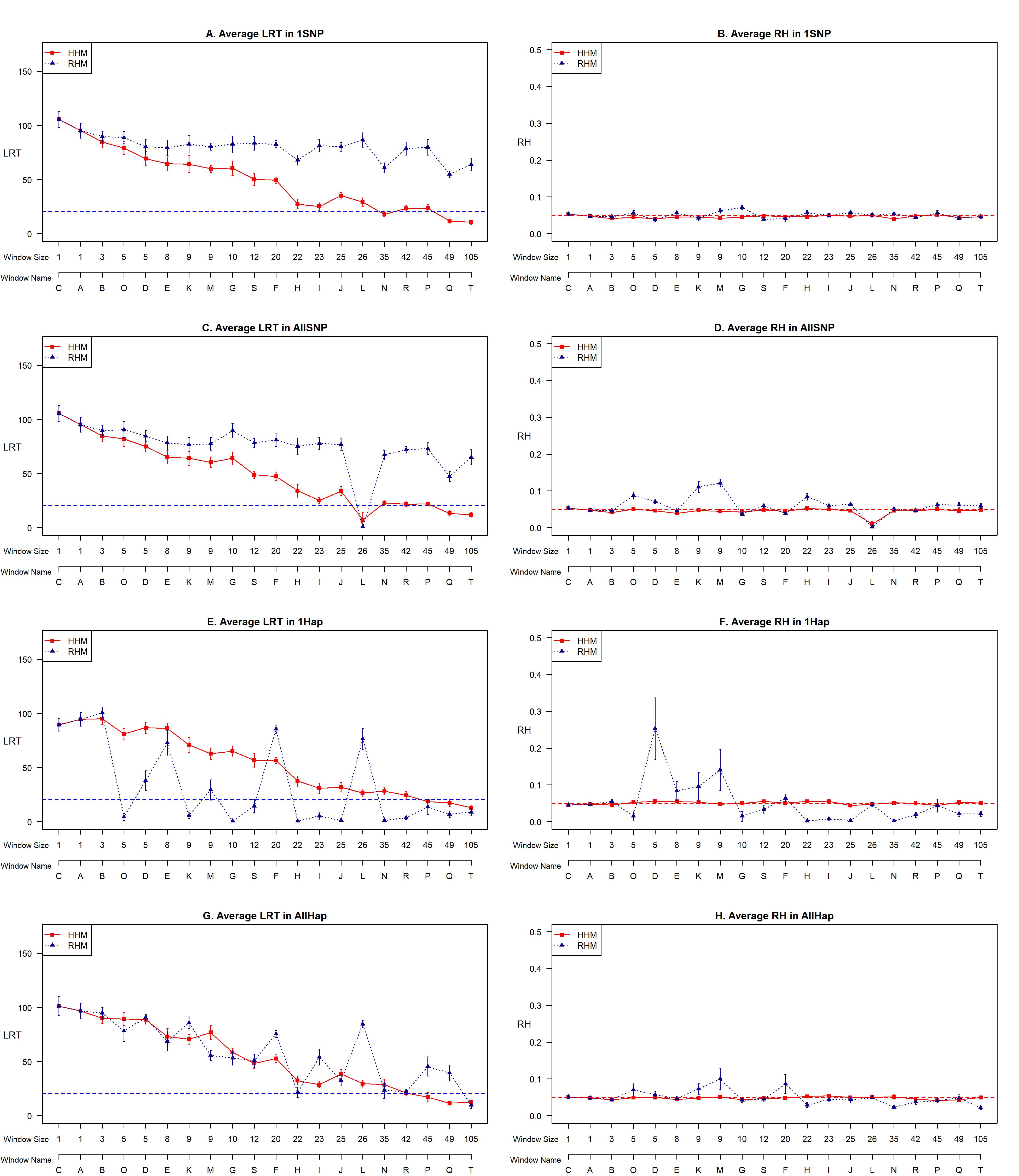


**Supplementary Figure S2: The ratio of averaged LRT (A,C,E,G) and RH (B,D,F,H) estimated by analysis windows based on 10cM/Mb recombination (T10) to the 5cM/Mb (T5) based windows by HHM and RHM methods against the ratio of the analysis window sizes for the 10cM/Mb (T10) to 5cM/Mb (T5) windows for each simulation scenario**


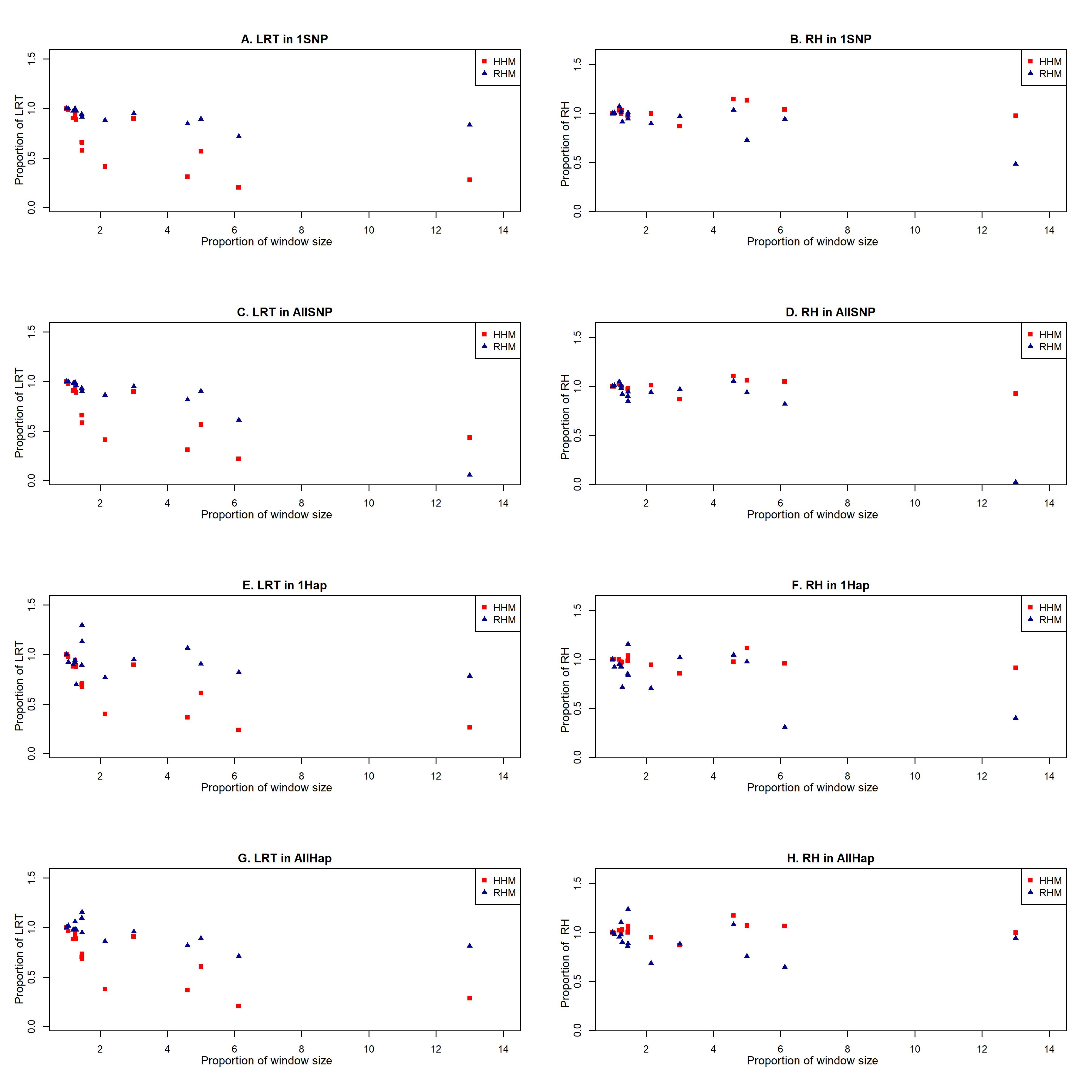


**Supplementary Figure S3: Standard Error of regional heritability estimates (SERH) against estimated regional heritability (RH) for all simulations in each method.** The red vertical dotted line represents simulated regional heritability (0.05) and the blue vertical solid line represents simulated trait total heritability (0.30).


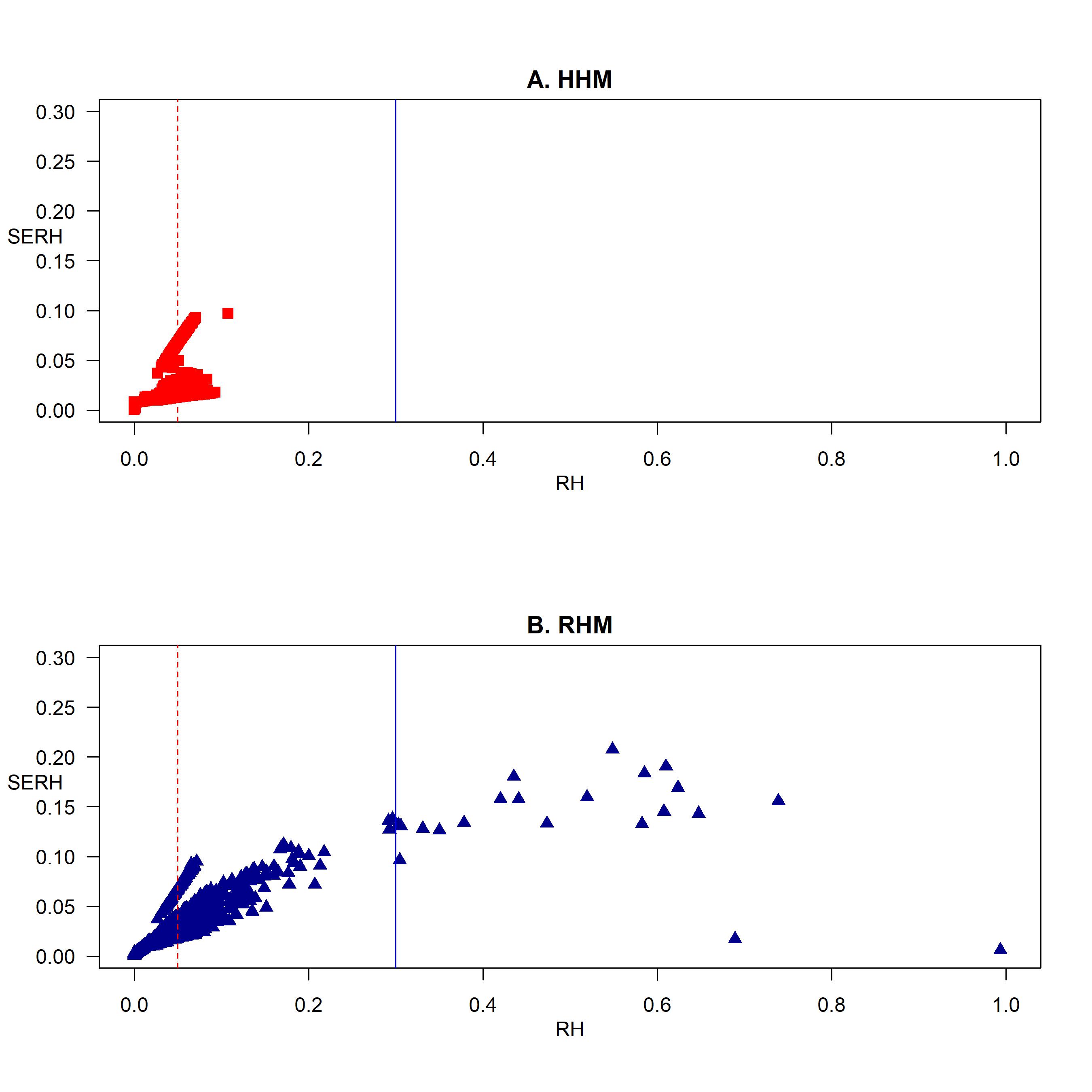


**Supplementary Figure S4: Plot of estimated RH against LRT for the each simulated scenario (1SNP, AllSNP, AllHap and 1Hap) by using the HHM (A,C,E,G) and the RHM (B,D,F,H) for all replicates of the simulated regions.** y-axis: estimated LRT (likelihood ratio test) is a statistical test to compare the goodness of fit of regional genomic relationship matrix in the model with the null model. x-axis: estimated RH (regional heritability) is proportion of trait variance explained by the regional genomic relationship matrix. The red vertical dotted line represents simulated regional heritability (0.05) and the blue vertical solid line represents simulated trait total heritability (0.30).


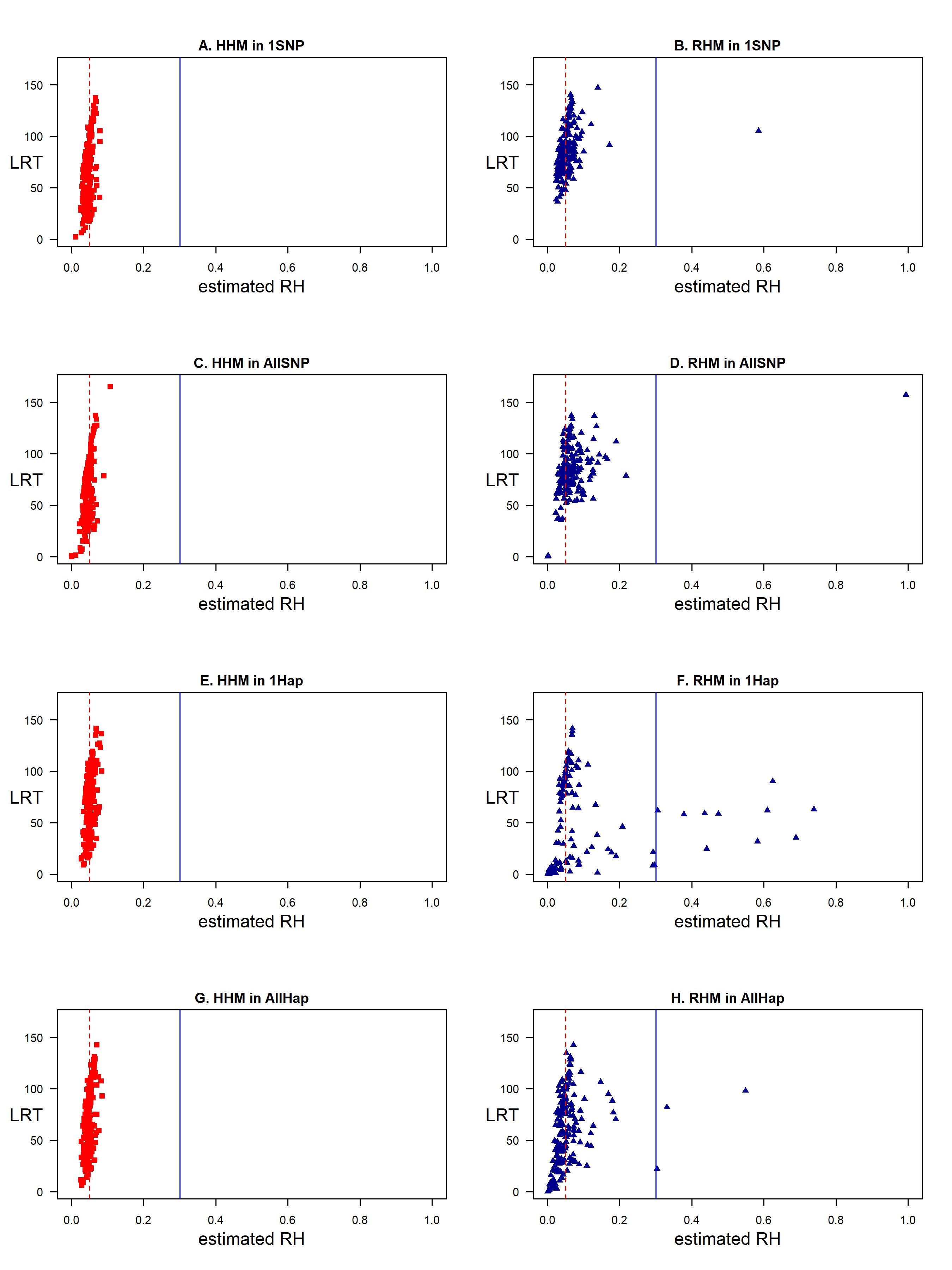

Supplement: Supplementary file 1 — Supplementary materials [file 41598_2018_23307_MOESM1_ESM.doc]
